# A Pilot Study on the Co-existence of Diabetes and Endometriosis in Reproductive-Age Women: Potential for Endometriosis Progression

**DOI:** 10.1007/s43032-023-01190-3

**Published:** 2023-02-14

**Authors:** Iad Alhallak, Charles M. Quick, Garrett L. Graham, Rosalia C. M. Simmen

**Affiliations:** 1 Department of Physiology & Cell Biology, University of Arkansas for Medical Sciences, Little Rock, AR USA; 2 Department of Pathology, University of Arkansas for Medical Sciences, Little Rock, AR USA; 3 The Winthrop P Rockefeller Cancer Institute, University of Arkansas for Medical Sciences, Little Rock, AR USA

**Keywords:** Endometriosis, Diabetes, Estrogen receptor, Progesterone receptor, Co-morbidity

## Abstract

Endometriosis (ENDO) is a chronic estrogen-dependent gynecological condition that affects reproductive-age women, causing pelvic pain, infertility, and increased risk for ovarian cancer. Diabetes mellitus (DM) is a metabolic disease with significant morbidity and mortality and rising incidence worldwide. The occurrence of DM among ENDO patients remains understudied, despite commonalities in these conditions’ immune, inflammatory, and metabolic dysfunctions. This pilot study evaluated whether a subset of women with ENDO manifests DM co-morbidity and if so, whether DM promotes ENDO status. Archived ectopic lesions obtained at ENDO surgery from non-diabetic (ENDO-N; *n* = 11) and diabetic (ENDO-DM; *n* = 15) patients were identified by a search of an electronic health database. Retrieved samples were analyzed by immunohistochemistry for markers of proliferation (Ki67, PTEN), steroid receptor signaling (ESR, PGR) and macrophage infiltration (CD68). Immunostaining data were expressed as percentages of immune-positive cells in lesion stroma and epithelium. In lesion stroma, the percentages of nuclear immune-positive cells were higher for ESR2 and lower for PGR-T, in ENDO-DM than ENDO-N patients. The percentages of nuclear immune-positive cells for ESR1 and PTEN tended to be higher and lower, respectively, in ENDO-DM than ENDO-N groups. In lesion glandular epithelium, the percentages of nuclear immune-positive cells were higher for ESR1 and ESR2, in ENDO-DM than ENDO-N groups. ENDO-N lesions had lower percentages of stromal CD68 immune-positive cells than ENDO-DM Type 1 lesions. Findings demonstrate DM in a subset of women with ENDO, which was associated with significant changes in lesion stromal and epithelial nuclear steroid hormone receptor levels, suggestive of disease progression.

## Introduction

Endometriosis (ENDO) is a condition characterized by the presence of uterine endometrial-like epithelium and stroma in extra-uterine sites, causing debilitating pelvic pain, dysmenorrhea, and infertility in 50% of afflicted women, and with an annual economic burden of ~ 50B in the USA alone [1]. While ENDO is considered benign in its initial stage, a history of ovarian/tubal ENDO is an independent risk factor in two subtypes of ovarian cancer, namely, clear-cell and endometrioid [2, 3]. The pathogenesis of ENDO-associated ovarian carcinoma remains unclear and is an area of intense investigations [4, 5]. Nevertheless, recent findings have shown that endometriotic lesions without concurrent cancers contain cancer-associated somatic mutations including for *KRAS*, *PTEN*, *ARID1*, and *PIK3CA* [6, 7]. The latter suggests that ovarian endometriotic lesions can progress to malignancy, given the proper signals and context.

Diabetes is a progressive disease affecting over 400 million people worldwide [8]. Type 1 diabetes mellitus (T1DM) and type 2 diabetes mellitus (T2DM) are characterized by significant genetic predispositions and shared dysfunctions in glucose homeostasis, resulting in elevated blood glucose levels in affected individuals. These conditions have distinct pathogenesis: T1DM results from the autoimmune destruction of pancreatic β-cells and hence, early progressive loss of insulin production while T2DM, a condition increasing with age, stems from the loss of insulin sensitivity of target cells, leading to defects in glucose clearance [9, 10]. T1DM appears first in children and adolescents, and while its adult-onset is increasingly diagnosed, the basis for the delayed manifestation is not well-understood [11]. T2DM is significantly affected by lifestyle factors (diet, physical activity, alcohol, and tobacco use), is preventable, and is generally manageable by lifestyle modifications [12]. Similar to ENDO [13, 14], T1DM and T2DM manifest significant inflammatory, immune, and metabolic dysfunctions and are associated with increased risk and progression of ovarian cancer [15–17].

There is no known predominance of ENDO in women with either T1DM or T2DM in the general population; however, women (and young girls upon initiation of menses) may suffer unknowingly from co-morbid conditions throughout their reproductive years since ENDO is difficult to diagnose, identify, and treat [1]. Importantly, because ENDO and DM are chronic conditions, women with co-morbidities are anticipated to have lower quality of life, may develop drug-drug interactions leading to reduction in treatment efficacies for each condition, and may face advancement in ENDO status.

The present investigation constitutes a pilot study to evaluate whether a subset of women with ENDO may suffer DM co-morbidity and if so, whether DM may progress ENDO. Using archived FFPE ectopic (ovarian/tubal) lesions from women with ENDO alone and with co-incidence of DM (T1DM or T2DM), we report herein that DM and ENDO may co-exist in reproductive-age women and that DM association in women with ENDO confers significant changes in lesion nuclear steroid hormone receptor levels, suggestive of increased estrogen dependency and heightened progesterone resistance, both of which constitute markers of endometriosis progression.

## Materials and Methods

### Sample Tissue Collection

The study was approved by the Institutional Review Board of the University of Arkansas for Medical Sciences (UAMS IRB#205,177). The Arkansas Clinical Data Repository, which is affiliated with the TriNetX health research platform, was used to identify female patients (20–60 years old inclusive) diagnosed from 2015 to 2019, using the search words “Endometriosis with no diabetes (ENDO-N),” “Endometriosis with Type 1 diabetes (ENDO-T1DM),” and “Endometriosis with Type 2 diabetes (ENDO-T2DM).” De-identified referral numbers for patients meeting the specific criteria were sent to the UAMS Department of Pathology and corresponding FFPE sections in the storage inventory, when available, were retrieved by our team pathologist (CMQ). The patient data (age and BMI at ENDO surgery, race/ethnicity, presence or absence of ovarian mass, use of progesterone for ENDO management) were subsequently obtained for all analyzed samples.

### Immunohistochemistry

FFPE samples were sectioned (5 μm) and processed by heat-induced epitope retrieval (citrate buffer) and subsequent incubation with designated antibodies as previously described [18]. Table 1 provides the list of the primary antibodies with their unique Research Resource Identifier (RRID), used at working dilutions following the RRID information (antibody registry.org) at incubation conditions of 4 °C for 16–24 h. Immunoreactivity was detected using the Vectastain Elite ABC kit (Vector Laboratories) and biotinylated anti-rabbit secondary antibodies (Vector Laboratories), and slides were counterstained with hematoxylin. The stained slides were digitized using the Leica Digital Pathology Whole Slide Scanner (Aperio Image Scope). Cells were scored as non-staining (i.e., only background staining) based on sections that were processed in parallel with the omission of the primary antibody. For each antibody-treated tissue section, a total of ~ 100 cells for each glandular epithelial and stromal compartment in 3–4 random fields were counted for numbers of nuclear-staining and non-staining cells. Analyses for CD68-immune-staining followed the same procedure except that stromal non-nuclear (cytoplasmic/membrane) staining cells were counted. Data are expressed as the percentages of nuclear-stained (Ki67, PTEN, ESR1, ESR2, PGR-T, PGR-B) or cytoplasmic/membrane-stained (CD68) cells, relative to the total number of cells counted.

**Table 1:** Antibodies used for IHC

Protein	Vendor/catalog number	RRID^1^	Working dilution
Estrogen receptor α	Santa Cruz/sc542	AB_63140	1:250
Estrogen receptor β	Millipore Sigma/05–824	AB_310195	1:200
Ki67	Abcam/Ab16667	AB**_**302459	1:200
Progesterone Receptor (total)	Santa Cruz/sc7208	AB_2164331	1:200
Progesterone Receptor-B	Cell Signaling/3157S	AB_2252606	1:400
Phosphatase and Tensin homologue	Cell Signaling/138G6	AB_823618	1:200
Macrophage CD68	Thermo Fisher Scientific/MA5-13,324	AB_10987212	1:200

^1^Research Resource Identifier (antibodyregistry.org)

### Data Analysis

Data were analyzed by the Shapiro–Wilk’s test for normality and compared for statistical significance of difference between experimental groups using the Mann–Whitney *U* test. The statistical tests were performed using the GraphPad Prism software (version 6). Data are presented as box plots indicating the upper and lower quartiles, range, and median (middle line) with whiskers indicating the maximum and minimum points. A *p* value ≤ 0.05 was considered to be significant. Principal component, multivariate regression, and binary logistic regression analyses were used to analyze the associations between the patient variables (age at surgery, race, progesterone usage, and BMI) with the protein biomarkers in glandular epithelial and stromal compartments.

## Results

### Patients’ Demographic Information

**Fig. 1:**
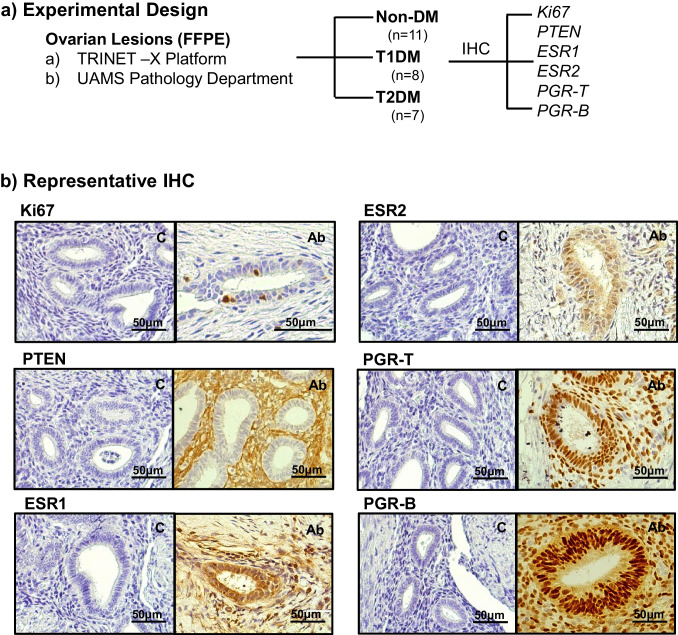
Analyses of endometriotic lesions. **a**. Schematic of tissue retrieval and analyses. Tissue samples for the study were identified from search of the Arkansas Clinical Data Repository, using the TriNetX health research platform, and retrieved from the UAMS Department of Pathology repository. Formalin-fixed paraffin-embedded sections were subjected to immunohistochemistry using the listed antibodies (Table 1). **b** Representative H&E-stained sections of lesions from non-diabetic women with ENDO. For each section stained with the indicated antibody (anti-Ki67, anti-PTEN, anti-ESR1, anti-ESR2, anti-PGR-T, anti-PGR-B), a corresponding section was processed in parallel in the absence of antibody to serve as control (labeled C)

Figure 1a provides a schematic summary of sample retrieval and subsequent analyses of tissue sections by immunohistochemistry (IHC). FFPE-processed blocks from women with ENDO without DM (*n* = 11), with TIDM (*n* = 8), and with T2DM (*n* = 7) were identified from surgical pathology reports of the Arkansas Clinical Data Repository based on a web-based search of the TriNetX Health Research Platform for the period covering 2015–2019. Medical records were de-identified and available FFPE sections at the UAMS Department of Pathology storage inventory were retrieved by our team pathologist (CMQ). Lesions were largely tubal and ovarian (with an exception of one omental) and classified as stages 3–4, based on the American Society of Reproductive Medicine guidelines [19]. Because of the small sample sizes of the ENDO-T1DM and ENDO-T2DM groups, these sets were combined and designated as ENDO-DM (Table 2). The mean age (years, y) of women undergoing surgery for removal of ENDO lesions was higher (*p* < 0.001) for patients without diabetes (ENDO-N; 41.9 ± 0.8 y) when compared to those with diabetes (ENDO-DM; 31.6 ± 1.8 y). Mean BMI (kg/m^2^) was higher at surgery (*p* = 0.007) for ENDO patients with DM (45.0 ± 3.4) than without DM (ENDO-N; 31.4 ± 2.7). The duration of progestin use for each group could not be determined due to lack of documentation. However, the numbers of patients within each group using progestin for ENDO treatment were comparable (*p* = 0.86). Similarly, there was no race/ethnic disparity in the patient population between the ENDO-N and the ENDO-DM groups (*p* = 0.19). Corresponding ovaries for all patients did not contain ovarian carcinoma, as reported from pathology records and a review of the surgical pathology slides (Table 2).

**Table 2:** Patient demographics

Race/Ethnicity	Age at ENDO Surgery	BMI at ENDO Surgery	Ovarian Mass	Progesterone
ENDO-N				
Black/African American	43	25.0	No	No
Black/African American	43	46.2	No	Yes
White/Caucasian	38	39.5	No	Yes
Black/African American	39	21.6	No	Yes
Black/African American	44	22.5	No	Yes
Black/African American	45	29.9	No	No
Black/African American	43	29.5	No	Yes
Black/African American	43	18.2	No	Yes
Black/African American	45	36.9	No	No
Black/African American	39	38.3	No	Yes
Black/African American	39	38.3	No	Yes
Mean ± SEM	41.9 ± 0.8	31.4 ± 2.7		
ENDO-DM				
White/Caucasian	24	33.2	No	Yes
White/Caucasian	25	39.7	No	Yes
Black/African American	34	36.5	No	Yes
White/Caucasian	24	33.1	No	Yes
Black/African American	28	52.8	No	No
Black/African American	29	61.1	No	No
Black/African American	31	52.4	No	No
White/Caucasian	26	21.6	No	No
Black/African American	40	52.9	No	No
Black/African American	29	61.8	No	Yes
Black/African American	36	24.2	No	No
Black/African American	44	46.2	No	Yes
Black/African American	26	61.8	No	Yes
Black/African American	34	49.8	No	No
Hispanic/Latino	44	48.2	No	Yes
Mean ± SEM	31.6 ± 1.8*	45.0 ± 3.4*		

^*^t-test (*p* ≤ 0.05, compared to ENDO-N)

### Immunohistochemistry

In a previous report [18], we showed that Ki67, PGR-T, ESR1, and ESR2 constitute valid biomarkers for endometriosis progression since their levels and patterns of nuclear immunopositivity in ectopic lesions differed significantly from those of corresponding eutopic endometria or non-diseased endometria. PTEN protein levels in lesions were additionally evaluated in the present study, given the protein’s anti-proliferative/pro-apoptotic actions and the reported PTEN mutations in ovarian endometriosis lesions [20]. Similar analyses for PGR-B were performed since disruption of PGR-B expression has been demonstrated in many uterine disorders including endometriosis [21–23]. A composite of representative immunostaining of lesions from women with ENDO-N, using specific antibodies to each protein, is shown in Fig. 1b. Nuclear staining for all proteins was demonstrated in both stromal and epithelial compartments.

### Stromal Immunoreactivity in ENDO Lesions with Diabetes Status

**Fig. 2:**
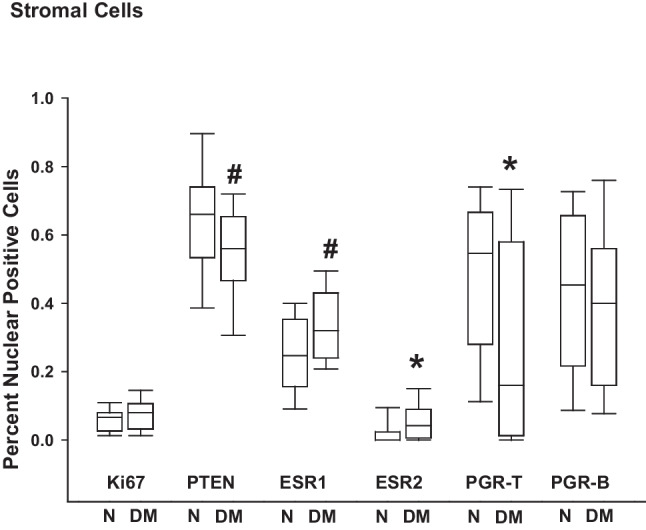
Stromal immunoreactivities of ENDO markers in ectopic lesions of women with and without diabetes. Tissue sections from non-diabetic women with ENDO (N) and from women with ENDO and diabetes (DM) were processed for immunohistochemistry, using specific antibodies as described under “Materials and Methods.” The percentages of nuclear-localized Ki67, PTEN, ESR1, ESR2, PGR-T, and PGR-B in lesion stromal cells were determined by counting the number of immunopositive-staining nuclei over the total number of cells counted × 100. For each tissue section, 3–4 random visual fields representing a total of ~ 100 cells were assessed. Data represent the percentages of nuclear immunopositive cells from 6 to 10 individual samples per group and are presented as box plots indicating the upper and lower quartiles, range, and median (middle line) with whiskers specifying the maximum and minimum points. *P ≤ 0.05; ^#^0. 10 ≤ *P* ≤ 0.05 between N and DM

Stromal cells of ENDO lesions from women without DM displayed varying levels of nuclear-localized immunoreactivities (expressed as percent of nuclear-positive cells) for the evaluated ENDO biomarkers (Fig. 2). In ENDO-N lesions, the highest percent immunoreactivities were seen for PTEN, PGR-T, and PGR-B, with Ki67 and ESR2 immunoreactivities displaying the lowest levels. The co-occurrence of DM in women with ENDO increased and decreased, respectively, nuclear-localized stromal immunoreactivities for ESR2 (*p* = 0.04) and PGR-T (*p* = 0.05), relative to those of women without DM. Stromal cells of ENDO lesions with DM tended to show reduced (*p* = 0.07) and increased (*p* = 0.06) nuclear PTEN and ESR1 immunoreactivity levels, respectively, relative to those of women without DM. Levels of nuclear immunoreactivities for Ki67 and PGR-B were not affected by DM status (Fig. 2).

### Glandular Epithelial Immunoreactivity in ENDO Lesions with Diabetes Status

**Fig. 3:**
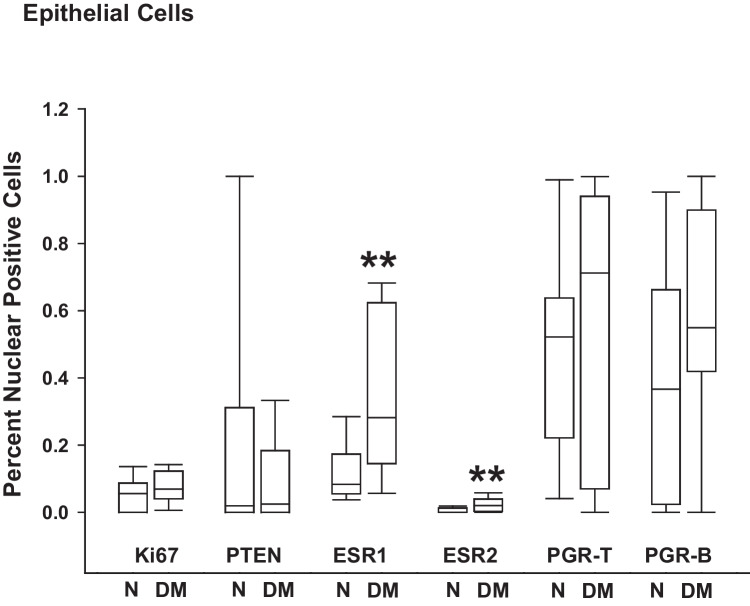
Glandular epithelial immunoreactivities of ENDO markers in ectopic lesions of women with and without diabetes. Tissue sections from non-diabetic women with ENDO (N) and from women with ENDO and diabetes (DM) were processed for immunohistochemistry, using specific antibodies as described under “Materials and Methods.” The percentages of nuclear-localized Ki67, PTEN, ESR1, ESR2, PGR-T, and PGR-B in lesion glandular epithelial cells were determined by counting the number of immunopositive-staining nuclei over the total number of cells counted × 100. For each tissue section, 3–4 random visual fields representing a total of ~ 100 cells were assessed. Data represent the percentages of nuclear immunopositive cells from 6 to 10 individual samples per group and are presented as box plots indicating the upper and lower quartiles, range, and median (middle line) with whiskers specifying the maximum and minimum points. **P ≤ 0.005 between N and DM

Glandular epithelial cells of ENDO lesions from women with DM displayed nuclear-localized immunoreactivities for ESR1 (*p* = 0.001) and ESR2 (*p* = 0.005) that were higher than those from women without DM (Fig. 3). By contrast, nuclear immunoreactivities for Ki67, PTEN, PGR-T, and PGR-B in these cells were not affected by DM status.

### Macrophage Biomarker CD68 Immunoreactivity in ENDO Lesions

**Fig. 4:**
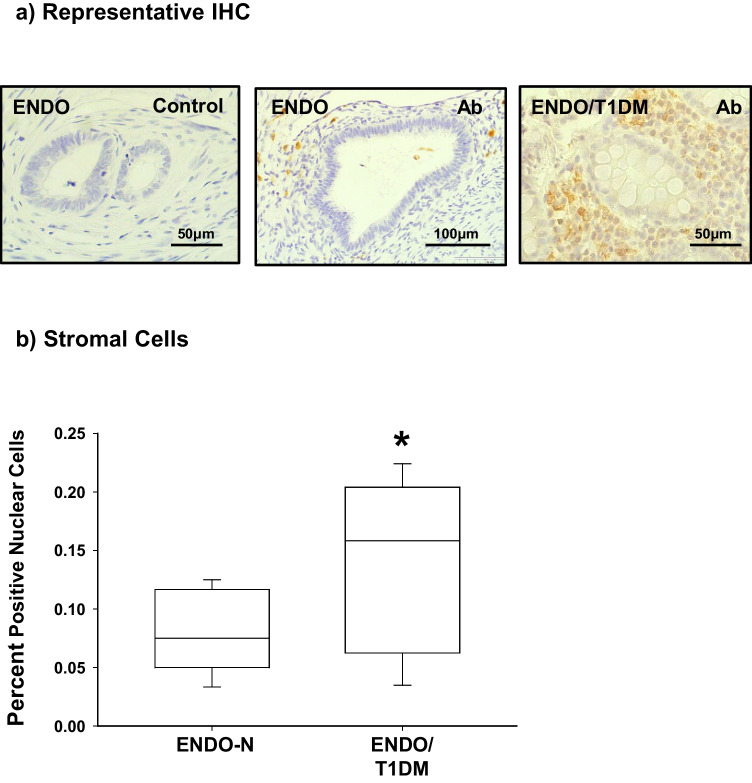
Monocyte/macrophage CD68 protein localization in ectopic lesions of women with and without Type 1 diabetes. Tissue sections from non-diabetic women with ENDO (ENDO-N) and from women with ENDO and type 1 diabetes (ENDO-T1DM) were processed for immunohistochemistry, using anti-CD68 antibody as described under “Materials and Methods.” **a** Representative images of anti-CD68 immunostaining for ENDO-N (middle panel) and for ENDO-T1DM (right panel) sections are shown. Left panel is an ENDO-N section processed in parallel in the absence of antibody. **b** For each tissue section, 3–4 random visual fields representing a total of ~ 100 stromal cells were assessed. Data represent the percentages of immunopositive stromal cells from 5 individual samples per group and are presented as box plots indicating the upper and lower quartiles, range, and median (middle line) with whiskers specifying the maximum and minimum points. *P ≤ 0.05 between ENDO-N and ENDO-T1DM

Macrophage infiltration of ovarian endometriomas has been previously reported [24]. Furthermore, we have shown in a mouse model of endometriosis that progression of ENDO in ectopic lesions with high fat diet was associated with increased localization of macrophages in stromal cells as measured by F/480 immunostaning [25]. Here, we used the human macrophage/monocyte selective biomarker CD68 to evaluate potential changes in macrophage infiltration of ENDO-N relative to ENDO-T1DM lesions. The limited availability of ENDO-T2DM lesions precluded parallel analyses of these samples. ENDO lesions stained positive for CD68 preferentially in cytoplasmic/membrane-associated compartments of lesion stromal cells (Fig. 4a). Immunostaining levels in stromal cells were higher for ENDO-T1DM than ENDO-N (Fig. 4b).

## Discussion

Endometriosis (ENDO) and diabetes (DM) individually affect women of reproductive age, yet the occurrence of ENDO and DM co-morbidity and its potential contribution to ENDO progression remain unexplored. In this pilot study, we provide clinical data to show the co-existence of ENDO in a subset of women with DM (T1DM and T2DM). Further, we demonstrate that DM status in ENDO women confers significant changes in steroid hormone receptor levels in lesion stromal and glandular epithelial compartments, relative to those of ENDO women without DM. Progesterone resistance, estrogen-dependency, and immune activation are hallmarks of ENDO development and progression. Reductions in levels of stromal ligand-bound PGR exacerbate ENDO status [18, 26, 27] and are associated with resistance to progestin therapy in women with ENDO [28]. Moreover, enhanced estrogen signaling leads to heightened cell inflammation mediated by ESR2 [29] and promotes cell proliferation mediated by ESR1 [30]. Here, we show that nuclear levels of PGR-T (stroma) were decreased while those of ESR1 (epithelia) and ESR2 (epithelia, stroma) were increased, in ENDO-DM relative to ENDO-N lesions. Furthermore, we found increased macrophage localization (CD68 immunoreactivity) indicative of immune activation known to be associated with ENDO progression [1, 25], in lesion stromal cells of ENDO women with Type I DM, relative to those of ENDO-N women. The tending decrease in lesion stromal PTEN immunoreactivity with DM status is consistent with earlier reports that subtle reductions of PTEN expression level are sufficient to promote cell proliferation and hence, cancer susceptibility [31]. Unexpectedly, there was a lack of coincident increases in the levels of Ki67 immunoreactivities in both lesion stromal and epithelial cells with DM status. We suggest that this may reflect in part the relatively advanced endometriotic stage of the lesions analyzed in the present study (largely stages 3–4) and the previously documented inability of Ki67 dynamics to accurately capture cellular proliferation index [32]. Multivariate analysis (data not shown) showed no significant association of patient variables of progesterone usage, race, BMI, and age of ENDO surgery with the evaluated protein biomarkers in lesion epithelial and stromal compartments, suggesting diabetes status as the major driver in the noted differences in these proteins’ expression levels.

In this study, the tissue samples were retrieved from patients in the age range of 20–60 years because ENDO symptoms (i.e., pelvic pain, heavy menses) are pronounced around age 20 (although the condition may initiate earlier) and recurrence in affected women may extend beyond the menopausal period. Interestingly, the study participants in the ENDO-N group were notably older than those of the ENDO/DM group. The significance of this finding is unclear since the data were obtained at ENDO surgery and not during initial diagnoses. However, we speculate that the earlier age of ENDO surgery for ENDO-DM patients may reflect their greater degree of pain/discomfort. The relationship between pain severity in ENDO women and DM status merits further investigation. The linkage of adiposity (measured by BMI) and endometriosis is complex and may be dependent on disease severity [33]. The higher BMIs shown for ENDO patients with DM align with the positive association of BMI and diabetes mellitus [34, 35]. By contrast, epidemiological studies indicate a negative association for obesity and ENDO progression [36, 37]. Nevertheless, since obesity does not protect against endometriosis [38] and in mouse models of the disease, high-fat diet induced obesity and inflammation increased endometriosis development [25, 39], there is a need for further evaluation of this relationship.

Despite the significant health and economic challenges imposed individually by DM and ENDO in female patients, the prospects of their co-incidence in a subset of women have not been assessed. Indeed, no work to date has clinically addressed whether DM status promotes ENDO and if ENDO treatments may complicate glycemic control in women with DM [40]. In a recent prospective study using data from the Nurses’ Health Study, Missmer and colleagues [41] reported no overall increased risk of T2DM for women with ENDO. However, the reverse relationship, whether DM promotes ENDO, has not been studied. Our pilot study, despite small sample sizes, provides support for further consideration of this possibility.

We acknowledge several limitations in the present study — these include small sample numbers, lack of ethnic diversity which does not allow for generalizability of results in the population, missing information on onset of DM status and on initial ENDO diagnoses, and lack of consideration on the possibility of other pre-existing/underlying disease in patients. Due to the small sample sizes, ENDO patients with T1DM or T2DM were analyzed as one group relative to ENDO patients with no DM. Given the distinct pathogenesis of T1DM and T2DM, future studies should consider the individual impact of T1DM vs T2DM on ENDO progression to inform screening or preventive interventions. A recent study showed that serum glucose levels were lower in ovarian ENDO patients than in healthy controls and that glucose together with those of inflammatory cytokine tumor necrosis factor-α, interleukin-6, and monocyte chemoattractant protein-1 may be useful as diagnostic serum biomarkers for staging of ENDO [42]. Since the participants in the reported study were not diabetic, the significance of the results in the context of our study is not clear. Nonetheless, these collective findings suggest that metabolic status may constitute a significant contributor to ENDO progression, consistent with the metabolic underpinnings of ENDO as suggested by us [25] and others [43]. Further research is merited to understand whether a potential feed-forward relationship between ENDO and DM exists and with relevance to glycemic control and other metabolic features in the patient population with co-morbidities.

In summary, while endometriosis is considered a largely benign disorder from a clinical perspective, co-morbidity with DM may lead to a progressive condition. Given that epithelial cells from endometriomas with no associated carcinoma express numerous cancer-associated mutations [7], effective management of ENDO and DM may constitute a promising strategy against the development of ovarian cancer. Moreover, a mechanistic understanding of a causal relationship between ENDO and DM may have implications for the treatment of ENDO in a subset of women with DM and for long-term glycemic control in patients with co-morbid DM and ENDO.

## Abbreviations

BMI: Body mass index
DM: Diabetes mellitus
ENDO: Endometriosis
ESR1: Estrogen receptor α
ESR2: Estrogen receptor β
FFPE: Formalin-fixed paraffin embedded
GE: Glandular epithelial
PTEN: Phosphate and tensin homolog
PGR-T: Progesterone receptor-total
PGR-B: Progesterone receptor-isoform B
ST: Stromal,
T1DM: Type 1 diabetes mellitus
T2DM: Type 2 diabetes mellitus
